# SNPest: a probabilistic graphical model for estimating genotypes

**DOI:** 10.1186/1756-0500-7-698

**Published:** 2014-10-07

**Authors:** Stinus Lindgreen, Anders Krogh, Jakob Skou Pedersen

**Affiliations:** Section for Computational and RNA Biology, Department of Biology, University of Copenhagen, Ole Maaloes Vej, 2200 Copenhagen, Denmark; Center of Excellence for GeoGenetics, Natural History Museum of Denmark and Department of Biology, University of Copenhagen, Oester Voldgade 5-7, 1350 Copenhagen K, Denmark; Biomolecular Interaction Centre, School of Biological Sciences, University of Canterbury, Private Bag 4800, 8041 Christchurch, New Zealand; Department of Molecular Medicine, Aarhus University Hospital, Skejby, Brendstrupgaardsvej 100, DK-8200 Aarhus N, Denmark

**Keywords:** Next-generation sequencing, SNP, Genotyping, Illumina, Ancient DNA

## Abstract

**Background:**

As the use of next-generation sequencing technologies is becoming more widespread, the need for robust software to help with the analysis is growing as well. A key challenge when analyzing sequencing data is the prediction of genotypes from the reads, i.e. correct inference of the underlying DNA sequences that gave rise to the sequenced fragments. For diploid organisms, the genotyper should be able to predict both alleles in the individual. Variations between the individual and the population can then be analyzed by looking for SNPs (single nucleotide polymorphisms) in order to investigate diseases or phenotypic features. To perform robust and high confidence genotyping and SNP calling, methods are needed that take the technology specific limitations into account and can model different sources of error. As an example, ancient DNA poses special challenges as the data is often shallow and subject to errors induced by post mortem damage.

**Findings:**

We present a novel approach to the genotyping problem where a probabilistic framework describing the process from sampling to sequencing is implemented as a graphical model. This makes it possible to model technology specific errors and other sources of variation that can affect the result. The inferred genotype is given a posterior probability to signify the confidence in the result. SNPest has already been used to genotype large scale projects such as the first ancient human genome published in 2010.

**Conclusions:**

We compare the performance of SNPest to a number of other widely used genotypers on both real and simulated data, covering both haploid and diploid genomes. We investigate the effects of read depth, of removing adapters before mapping and genotyping, of using different mapping tools, and of using the correct model in the genotyping process. We show that the performance of SNPest is comparable to existing methods, and we also illustrate cases where SNPest has an advantage over other methods, e.g. when dealing with simulated ancient DNA.

**Electronic supplementary material:**

The online version of this article (doi:10.1186/1756-0500-7-698) contains supplementary material, which is available to authorized users.

## Findings

We present a novel approach to the genotyping problem where a probabilistic framework describing the process from sampling to sequencing is implemented as a graphical model. This makes it possible to model technology specific errors and other sources of variation that can affect the result. The inferred genotype is given a posterior probability to signify the confidence in the result. SNPest has already been used to genotype large scale projects such as the first ancient human genome published in 2010.

## Introduction

There has been a revolutionizing development in sequencing technology from the first genome sequencing projects, which were initiated in the late 1980s, and up until today. Both the time frame and cost of sequencing have decreased significantly since then, and today a single research lab can generate billions of high-quality base pairs from millions of reads in a short time and for a reasonable price.

Next-generation sequencing (NGS) techniques cover a wide range of technologies that succeed the Sanger approach [1]. Common for NGS is the high-throughput nature of the technology where a large number of DNA templates is sequenced in parallel [2, 3]. However, all these new techniques have their own method specific biases that need to be addressed: The Roche 454 Genome Sequencer have problems with homopolymers that lead to increased insertion-deletion error rates, and for Illumina sequencing the signal-to-noise ratio degrades as a function of read length resulting in high error rates in the 3’ ends of reads [2].

NGS methods generate a large amount of data but the various sources of error need to be modeled for genotyping and SNP calling to work optimally. The SNP calling method has to be robust to noise and, preferably, not biased by the systematic errors in the NGS platform. Furthermore, since the read depth along the sequenced DNA template varies, the genotyper should be able to optimally use all information available, and to produce information about how reliable the inferred genotype is.

A new but growing field is the sequencing of ancient DNA, such as the woolly mammoth [4], Neanderthal [5] and ancient humans [6–8]. This presents special challenges such as a limited amount of data and hence lower coverage and read depth, but also fragmentation and post mortem damage of the DNA pose some unique problems. This has lead to the development of specific tools [9] and pipelines [10] for how to deal with this data, but a genotyper taking these peculiarities directly into account is still lacking. Interestingly, novel medical applications present some of the same challenges from samples that have been formalin-fixed and paraffin-embedded [11].

We present SNPest which models the genotyping and SNP calling from the raw read sequences in a fully probabilistic framework. The problem is described using a generative probabilistic graphical model [12]. There are many advantages in using a probabilistic model: The sampling and sequencing process is modeled explicitly which makes the approach flexible, all results get an intuitive confidence measure directly from the method, it can utilize all available information, and it is easily extended to take other sources of error or prior knowledge into account.

## Results

We have tested SNPest on both simulated and real data, and we compare its performance to a number of other widely used genotypers: GeMS [13] and FreeBayes [14], both of which are able to specifically model either haploid or diploid data, as well as mpileup combined with bcftools from the SAMtools package [15] and HaplotypeCaller from the GATK package [16–18], both of which only support diploid genomes.

Furthermore, whether the genotyper uses the reference allele as a prior when calling SNPs will affect the outcome. SNPest is able to either use or ignore the reference genome in the calculations, and we test both options in this paper. GeMS uses the reference allele to model the distribution over possible genotypes, and there is no parameter to turn this off. FreeBayes by default does not use the reference allele, but this can be changed when calling the program. We only use the default behavior in the paper. The SAMtools/bcftools method uses the reference allele in the process. GATK also uses the reference allele when genotyping the data.

Both SNPest and GeMS are able to specify the maximum number of reads used at each position in the genome. Obviously, limiting the number of nucleotides used will speed up the calculations at the risk of making wrong calls, but it also makes it possible to investigate how varying read depths affect genotype calls. We therefore tested both SNPest and GeMS using read depths of max 5, 10, 20, 30, 40, 50 and 60 on haploid data in order to investigate the robustness of both methods. For low depths, a certain variation in the number of SNPs called can be seen between runs due to the random down sampling of reads (i.e. when using less than the available data, a subset of reads is chosen randomly). FreeBayes does not have this feature and is only run using all the available data. SNPest is run both with and without using the reference genome as a prior. The other methods were run with default settings as described above.

The haploid reads were mapped to the reference genome using two different tools, Bowtie2 [19] and BWA-PSSM [20], using default settings. The mapped reads were processed using the mpileup command from SAMtools [15] with flags ‘-s’ to output the mapping qualities, ‘-q 25’ to only keep reads with mapping quality greater than 25, and ‘-Q 0’ to keep all bases irrespective of base quality.

When a genotyper is run on a data set, a large set of SNP candidates will be found. However, this set will normally not be used directly, and only a subset of these positions will be called as SNPs by the genotyper depending on the amount of evidence for the specific change. How to filter the SNP candidates to create a high confidence set of SNPs depends on the program being used. For each test we report the total number of SNP candidates reported by the genotyper (All) and a smaller, high-quality set (QC). For SNPest, this set consists of all SNPs where the posterior probability (reported as a Phred-like quality score [21]) is >30. For GeMS we use a threshold of 0.01 for the reported Dixon Q-test p-value as suggested by the authors (personal communication). Results from FreeBayes and GATK are filtered using a quality threshold of 30, and results from SAMtools are filtered using the command “vcfutils.pl varFilter” as described in the manual.

### Data sets

The real haploid data is generated from the REL606 *E. coli* strain [22] using 250 bp paired-end sequencing on a MiSeq to an average depth of 27X. The reference sequence for this strain is well-known, and no SNPs should be present when analyzing the mapped reads. As part of the test, we also examine the behavior of both SNPest, GeMS and FreeBayes in the presence of contaminating sequences in the data. This was done by analyzing the above data set both in its raw form and after removing residual adapter fragments with the AdapterRemoval program [23].

The simulated single-end ancient DNA data was generated based on the REL606 reference genome using ART [24]. A read length of 36 bp was used to simulate data to a depth of 27X of the *E. coli* genome, and no SNPs are simulated in the data. This resulted in 13,889,340 reads. These reads were furthermore mutated to yield an ancient DNA profile using a script provided by Peter Kerpedjiev (personal communication and [20]) based on the damage profile reported in [25]. This resulted in 1,884,087 reads being changed in one or more positions.

The diploid data used is from the 1000 Genome Project [26]. Instead of performing a full scale test on the whole human genome using all the different methods, we focus on two chromosomes and investigate the effects of read depth by including a low depth and a high depth data set. Since all methods are tested on the same smaller data sets, the relative performance can still be evaluated. Specifically, we use a low depth data set mapped to chromosome 20 using BWA [27] (sample HG00240, paired-end Illumina data, length 101 bp) and a high depth data set mapped to chromosome 22 using BWA (sample NA12878, paired-end Illumina data, length 100 bp). Both data sets were downloaded as re-aligned and re-calibrated bam-files.

### Performance on haploid data

In Table 1 we report the results from the cleaned *E. coli* data using the haploid mode of SNPest, GeMS and FreeBayes. In this case, we would not expect any SNPs since we are mapping the haploid reference genome to itself.

**Table 1:** **Performance of SNPest, GeMS and FreeBayes on real data**

	SNPest				GeMS				FreeBayes			
	Bowtie2		BWA-PSSM		Bowtie2		BWA-PSSM		Bowtie2		BWA-PSSM	
Depth	All	QC	All	QC	All	QC	All	QC	All	QC	All	QC
5	3	0	1	0	5	0	36	6				
10	0	0	0	0	5	0	32	2				
20	0	0	0	0	5	0	32	2				
30	0	0	0	0	5	0	32	2				
40	0	0	0	0	5	0	32	2				
50	0	0	0	0	5	0	32	2				
60	0	0	0	0	5	0	32	2	170	0	125	0

SNPest and GeMS use various fractions of the available data (from maximum 5 reads per site to maximum 60 (in this case all) reads per position), and FreeBayes is only run using all available data. The REL606 strain of *E. coli* was sequenced on the MiSeq platform to an average depth of 27X. Residual adapters were removed using AdapterRemoval, and the cleaned reads were mapped using two different mappers, Bowtie2 and BWA-PSSM. No SNPs are expected in this mapping, as we are mapping a known sequence back to itself. SNPest used the reference genome as a prior (see Additional file 1 for more results). All: All SNP candidates. QC: Number of SNPs after filtering on quality (SNPest and FreeBayes: Genotype quality of >30. GeMS: Dixon Q-test p-value <0.01).

SNPest only reports SNP candidates at the lowest read depth of 5X (3 for the Bowtie2 mapping, 1 for the BWA-PSSM mapping) and all are removed in the high quality set. As mentioned, FreeBayes was only run using all available data and predicts 125 to 170 SNP candidates, all of which are removed in the filtered data set. GeMS consistently reports 5 SNP candidates when using Bowtie2 as the mapper, of which none pass QC irrespective of read depth. When mapping with BWA-PSSM, between 32 and 36 SNP candidates are reported depending on read depth, of which 2-6 make it through QC. The results do not change significantly when analyzing the data where residual adapters have not been removed although more wrong SNPs are called as expected (see Additional file 1).

Using the simulated ancient DNA makes it possible to investigate how sensitive the tools are to specific patterns of errors in the data. We use a simulated data set similar to the one used above, i.e. haploid *E. coli* data with an average depth of 27X, but with a short read length of 36 bp and only single-end data. The reads were mapped using Bowtie2 and BWA-PSSM, but it should be noted that the latter tool was used without specifying an ancient DNA model and, thus, the mapping could be improved. However, the purpose of this test is to compare genotyping on different mappings and not to compare specific mapping tools.

In the context of ancient DNA, whether the reference genome is used or not has a larger impact than in the above test. In real applications (depending on the age of the sample), the reference might not be known but an extant related species is used as a scaffold instead. Whether or not to use the prior information in the reference genome for genotyping depends on the specific sample. As above, we have run SNPest with and without using the reference genome in the calculations, and we also compare results with and without using the specific model for ancient DNA. The results for SNPest (without using the reference) are shown in Table 2, and the results for GeMS and FreeBayes are shown in Table 3 (see Additional file 1 for additional results).

**Table 2:** **Simulated ancient DNA based on the REL606** ***E. coli*** **genome**

	SNPest, standard model				SNPest, damage model			
	Bowtie2		BWA-PSSM		Bowtie2		BWA-PSSM	
Depth	All	QC	All	QC	All	QC	All	QC
5	198	11	763	80	66	0	236	4
10	55	0	184	0	51	0	154	0
20	55	0	183	0	51	0	154	0
30	55	0	183	0	51	0	154	0
40	55	0	183	0	51	0	154	0
50	55	0	183	0	51	0	154	0
60	55	0	183	0	51	0	154	0

Read lengths of 36 bp and an average depth of 27X was simulated, and DNA damage was simulated in the reads as described in the main text. SNPest was run in the haploid mode, without using the reference genome, and both with and without the damage model.

**Table 3:** **Simulated ancient DNA based on the REL606** ***E. coli*** **genome**

	GeMS				FreeBayes			
	Bowtie2		BWA-PSSM		Bowtie2		BWA-PSSM	
Depth	All	QC	All	QC	All	QC	All	QC
5	88	32	298	97				
10	55	15	178	46				
20	55	15	177	46				
30	55	15	177	46				
40	55	15	177	46				
50	55	15	177	46				
60	55	15	177	46	2808	0	2680	0

Read lengths of 36 bp and an average depth of 27X was simulated, and DNA damage was simulated in the reads as described in the main text. GeMS and FreeBayes were both run in haploid mode, GeMS with varying maximum read depths, and FreeBayes using all data.

We first compare SNPest with and without explicitly modeling damage. It is seen that the number of SNP candidates does not change dramatically, although this will of course depend on the actual data set and the amount of damage. The total number of SNP candidates decreases somewhat going from the general error model (55-198 for Bowtie2 and 183-763 for BWA-PSSM) to the specific damage model (51-66 for Bowtie2 and 154-236 for BWA-PSSM). Thus, modeling damage does modestly decrease the number of SNP candidates, and the most pronounced difference is at the lowest depth of 5X where the total number of SNP candidates decreases by appr. 66%. In both cases we only predict high confidence SNPs at the the lowest 5X depth, and the number of SNPs in the high quality set is decreased substantially by using an explicit damage model (from 11 to 0 for the Bowtie2 mapping, and from 80 to 4 for the BWA-PSSM mapping). It is worth noting that most ancient DNA data sets would be in the very low average depth range. This is due to the nature of the data where the amount of endogenous DNA is very low and further limited by damage and fragmentation. This limits the amount of available DNA irrespective of read lengths and other technological advances.

Comparing SNPest to GeMS, we first note that the number of SNP candidates does not differ a lot between the two methods: GeMS has between 55 and 298 SNP candidates, depending on the mapping tool used. SNPest using the standard model has the largest number of SNP candidates at the lowest read depth (763 at 5X using BWA-PSSM). Introducing the damage model reduces the number of SNP candidates so SNPest consistently has fewer (although a similar number) than GeMS at all depths. However, the most significant difference is the number of predicted high confidence SNPs where it is clear that GeMS makes many more wrong calls than SNPest. GeMS predicts between 15 and 97 SNPs, and it is worth noting that GeMS predicts high confidence SNPs even at high read depths, indicating that the approach used in SNPest is well suited for biased data where the expected errors can be modeled. FreeBayes using all available data finds the highest number of SNP candidates (2,680 to 2,808), but all are removed in thefiltered set.

### Performance on diploid data

The performance on diploid data was evaluated for SNPest, GeMS, FreeBayes, GATK’s HaplotypeCaller, and SAMtools coupled with bcftools using the low depth and high depth data sets from the 1000 Genomes Project mentioned above. The test is carried out in a similar way to what was done in the GeMS paper [13]: The mapped data was analyzed using all genotypers, and the predicted high-confidence SNP sets (as defined above) were compared between the methods. Note that this means that SNPest only predicts SNPs and indels if at least 10 reads support the polymorphism.

For many reasons, genotyping a diploid genome from low depth data is much more challenging than genotyping a haploid genome. First, the number of possible genotypes is much larger (10 vs 4 possible combinations if strandedness is not considered). Second, the risk of sampling only one of the alleles at a heterozygous position increases with lower depth. Third, relying on the reference genome can bias the genotypes towards false heterozygote calls (if the true genotype is a homozygous SNP). Fourth, with lower depth and a diploid genome, errors in the data can have a proportionally higher impact. All this needs to be considered in the process.

We investigate a number of metrics to evaluate the quality of the predictions: The predicted SNP rate (i.e. the number of predicted SNPs divided by the number of positions); the overlap between predicted SNPs and annotated SNPs in dbSNP, version 139 [28]; the fraction of SNPs that are exclusively predicted by each method; the number of predicted insertions/deletions and the overlap with dbSNP (excluding GeMS); and the homozygous:heterozygous ratio for predicted SNPs. We also look at the overlap between each method and the set of SNPest predictions.

Since we do not know the correct answer for the data, and therefore cannot calculate sensitivity and specificity (or precision) directly, the above metrics are used to elucidate the performance of the different methods despite the shortcomings. The SNP rate can be compared between programs and related back to the expected rate. For the overlap between predicted SNPs and dbSNP it should be noted that dbSNP is not specific for the two chromosomes included in the test but contains common SNPs from the full human genome. Most SNPs from the two chromosomes that are annotated in dbSNP will not be present in the sequence data used here, and some SNPs that are present in the data might not be annotated in dbSNP. However, since dbSNP contains common SNPs it can be assumed that the majority of correct SNP predictions should be in the database (i.e. a large fraction of correctly predicted SNPs should overlap with dbSNP). It can also be assumed that true SNPs might be predicted by multiple programs, which indicates that the fraction of predictions that are exclusively found by one program should be relatively small. The homozygous:heterozygous ratio can show if there is a strong bias in one direction in the predicted SNPs. Previous studies have shown an expected ratio of approximately 0.8 [29]. To particularly investigate SNPest, we also look at the fraction of SNPest predictions that each of the other methods also predict, again assuming that a large overlap is a sign of quality. The overlaps in predictions between every pair of methods are presented in the Additional file 1.

**Figure 1:**
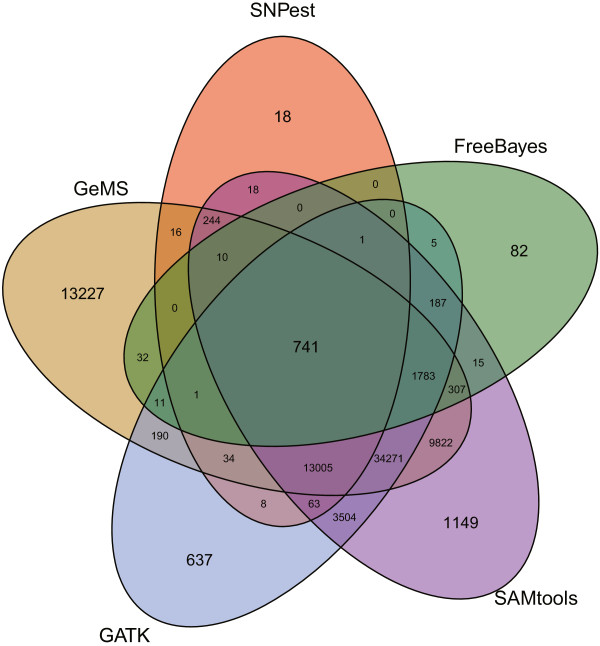
**Predicted SNPs on low depth diploid data from human chromosome 20.** The Venn diagram shows the performance of the five genotypers used (SNPest without using the reference genome, FreeBayes, SAMtools with bcftools, GATK’s HaplotypeCaller and GeMS) and illustrates the overlap in predicted SNPs between every combination of methods.

The low depth results are illustrated in the Venn diagram, Figure 1, showing the overlap in predicted SNPs between the five methods tested here, and tabulated in Table 4. The number of predicted SNPs varies from 3,175 (FreeBayes, SNP rate of 0.01%) to 73,694 (GeMS, SNP rate of 0.13%), with SNPest predicting 14,159 SNPs (a SNP rate of 0.02%). The relatively small number of SNPest predictions (with an expected SNP rate of 0.1%, see methods) is most likely due to the low amount of data causing the confidence in the predicted SNPs to decrease, resulting in more potential SNPs being filtered away. Especially the depth criteria (minimum 10X) means that many potential changes are not called. If a minimum depth of 5X was used instead, the number of SNPs increase to 36,140 (a SNP rate of 0.06%). However, the set of predicted SNPs shows a large overlap with dbSNP (99.42%, similar to GATK with 99.44%). Overall, the methods all show good concordance with dbSNP, with GeMS having the smallest overlap (87.59%).

**Table 4:** **Results on low depth, diploid data from human chromosome 20**

Program	#SNPs	SNP rate	dbSNP	SNPest	Excl.	Indels	dbSNP	Homo:hetero
SNPest	14,159	0.02%	99.42%	100.00%	0.13%	454	59.03%	0.64
FreeBayes	3,175	0.01%	98.90%	5.32%	2.58%	330	60.91%	1.12
SAMtools	65,120	0.11%	99.01%	99.46%	1.76%	6,918	60.18%	0.66
GATK	54,441	0.09%	99.44%	97.84%	1.17%	7,773	60.77%	1.09
GeMS	73,694	0.13%	87.59%	99.24%	17.95%	N/A	N/A	0.62

The results from SNPest (without using the reference genome), FreeBayes, SAMtools with bcftools, GATK’s HaplotypeCaller and GeMS are shown. For each method, we report the number of high quality SNPs, the SNP rate, the fraction overlap with dbSNP 139, the fraction of SNPest predictions in common, the fraction of exclusive SNPs only predicted by this method, number of insertions/deletions, fraction of insertions/deletions found in dbSNP 139, and homozygous:heterozygous ratio for SNPs.

Only 741 SNPs are predicted by all methods which is mainly due to the small number of SNPs from FreeBayes - an additional 13,005 SNPs are predicted by the remaining four methods. SNPest has the lowest number of SNPs only found by a single method (18 SNPs, 0.13% of predictions), with the other methods ranging from 82 (FreeBayes, 2.58%) to 13,227 (GeMS, 17.95%). Most of the SNPs predicted by SNPest are also found by the remaining methods (GATK predicts 97.84% of SNPest SNPs, GeMS 99.24%, and SAMtools 99.46% – FreeBayes only predicts 5.32% due to the low number of SNPs). The number of predicted indels varies a lot from 330 for FreeBayes to 7,773 for GATK, with SNPest predicting 454 indels. However, the overlap with dbSNP is around 60% for all methods which indicates very low sensitivity for Freebayes and SNPest. However, as mentioned above, if less than 10 reads support a mutation it is not part of the SNPest high confidence set. If the minimum depth requirement was changed to 5X, the number of predicted indels increases to 3,606.

The homozygous:heterozygous ratio for the predicted SNPs varies between methods, with SNPest, SAMtools and GeMS all having a ratio around 0.6, and GATK and FreeBayes having a ratio around 1.1 – i.e. on either side of the expected ratio. In low depth data, using the reference genome in genotyping can strongly affect the predicted SNPs by calling true homozygous SNPs as heterozygous (with the reference nucleotide as one allele). To test this, SNPest was also run in this mode (see Additional file 1 for results). The results are almost unchanged, except for the homozygous:heterozygous ratio which is dramatically affected with all SNPs now being called as heterozygous.

**Figure 2:**
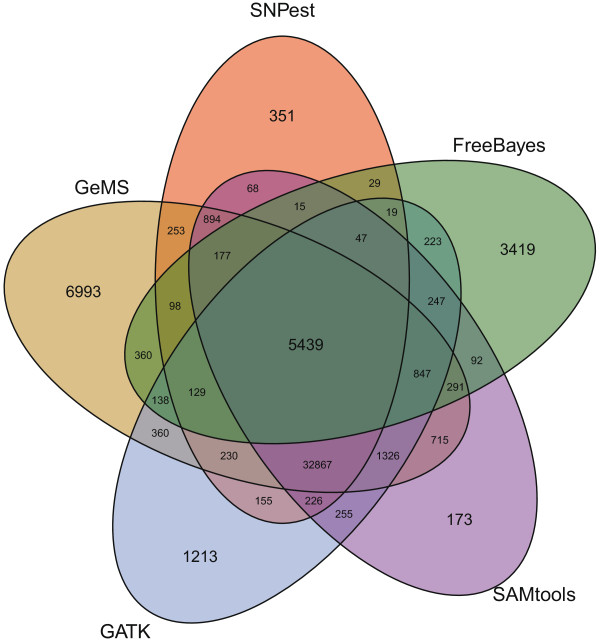
**Predicted SNPs on high depth diploid data from human chromosome 22.** The Venn diagram shows the performance of the five genotypers used (SNPest without using the reference genome, FreeBayes, SAMtools with bcftools, GATK’s HaplotypeCaller and GeMS) and illustrates the overlap in predicted SNPs between every combination of methods.

The results from the high depth data set are shown in Figure 2 and Table 5. FreeBayes again predicts the smallest number of SNPs (11,570, SNP rate of 0.03%) and this time also with the smallest overlap with dbSNP (87.99%). The remaining methods are in much better agreement, predicting from 40,997 SNPs (SNPest, SNP rate of 0.12%) to 51,117 SNPs (GeMS, SNP rate of 0.15%). SNPest, SAMtools and GATK all have a SNP rate of 0.12-0.13% and all have an overlap with dbSNP of around 99%, with only 89.22% of GeMS predictions overlapping with dbSNP. This SNP rate is as expected for SNPest, illustrating that the confidence in the predicted SNPs has increased leading to a larger high quality set. Changing the minimum depth to 5X only increases the number of SNPs to 42,140 (a SNP rate of 0.13%). There are 5,439 SNPs predicted by all five methods, but as before with a large number – 32,867 SNPs – predicted by all except FreeBayes.

**Table 5:** **Results on high depth, diploid data from human chromosome 22**

Program	#SNPs	SNP rate	dbSNP	SNPest	Excl.	Indels	dbSNP	Homo:hetero
SNPest	40,997	0.12%	99.17%	100.00%	0.86%	82	57.32%	0.46
FreeBayes	11,570	0.03%	87.99%	14.52%	29.55%	511	60.86%	4.67
SAMtools	43,679	0.13%	99.37%	96.92%	0.40%	3,880	57.45%	0.49
GATK	43,721	0.13%	99.29%	95.40%	2.77%	5,660	56.29%	0.58
GeMS	51,117	0.15%	89.22%	97.78%	13.68%	N/A	N/A	0.50

The results from SNPest (without using the reference genome), FreeBayes, SAMtools with bcftools, GATK’s HaplotypeCaller and GeMS are shown. For each method, we report the number of high quality SNPs, the SNP rate, the fraction overlap with dbSNP 139, the fraction of SNPest predictions in common, the fraction of exclusive SNPs only predicted by this method, number of insertions/deletions, fraction of insertions/deletions found in dbSNP 139, and homozygous:heterozygous ratio for SNPs.

As before, the other methods also predict most of the SNPs found by SNPest, from 95.40% to 97.78%, with FreeBayes again finding the lowest number of SNPs and therefore also only an overlap of 14.52%. The number of SNPs found by only a single method varies from 173 (SAMtools, 0.40% of predictions) to 6,993 (GeMS, 13.68%), with 29.55% of FreeBayes predictions being found by no other method. SNPest predicts 351 SNPs, or 0.86% of all its predictions, not found by any other method. As before, the number of predicted indels varies a lot from 82 (SNPest) to 5,660 (GATK) but with an overlap with dbSNP around 57% for all methods. Changing the minimum depth to 5X for SNPest increases the number of indels to 2,841 indicating that a more relaxed filtering of indels might be better.

The homozygous:heterozygous ratio is similar to what we saw for chromosome 20, with SNPest, SAMtools and GeMS finding around twice as many heterozygous than homozygous SNPs, but this time joined by GATK with a similar ratio. FreeBayes is the outlier with almost five times as many homozygous than heterozygous SNPs. In this case, using the reference genome has a much smaller impact on SNPest predictions because of the amount of observations per site, and the ratio changes from 0.46 to 0.31 (see Additional file 1).

## Discussion

We have presented SNPest, a probabilistic tool for genotyping next-generation sequencing data. SNPest can model various biases in the data, and it reports a quality score in Phred format giving the posterior probability of the given genotype. This is a useful metric to filter the SNP candidates and generate high-quality sets for further analysis. We have shown that SNPest performs favorably to other available genotypers on both real and simulated data. In all the haploid test sets, SNPest only makes a few wrong SNP calls, namely when using the lowest read depths and having residual adapter sequences in the reads. Otherwise, SNPest makes no wrong high-quality SNP calls. On the other hand, GeMS makes more albeit still few wrong SNP calls both in the clean data and when residual adapter sequences are present. In the ancient DNA test set, the difference is more pronounced and the strength of SNPest is seen.

In the diploid test, we show that the predictions made by SNPest show a good overlap with known SNPs in dbSNP, and that the majority of the predictions overlap with the other methods tested here. If the reference genome is used on low depth data, the model seems to overestimate heterozygote SNPs which is expected given the model used and the data available. This is not a problem in the high depth data where the extra observations help resolve the issue. The indel predictions made by SNPest also show good overlap with annotated indels in dbSNP similar to the predictions made by other programs but less restrictive filters might improve the results.

SNPest is implemented in such a way that novel models of errors or sample specific biases can easily be used. The probability matrix used when translating quality scores to probabilities has to be modified and the resulting files have to be named as described in the README file. Then, the specified model can be used by simply passing the model name when calling SNPest.

## Methods

Using sequencing data, you can either perform mapping, where the reads are aligned to a reference genome, or *de novo* assembly, where the reads are combined without a reference to infer the original template DNA sequence. For the task of genotyping, the major difference between the two is whether a reference genome is available or not, and whether this information should be used when inferring the genotype of the sequenced organism. Furthermore, most next-generation sequencing platforms produce a string of quality scores, normally encoded as Phred scores [21], that indicate the probability that the reported base is actually correct. This information should be included in the genotyping procedure.

There are a number of biases affecting the sequencing process and subsequent mapping [30, 31]: The quality scores are not evenly distributed but tend to drop off towards the 3’ end of the read. This results in more wrongly called bases occurring in the 3’ end of reads. Furthermore, the read depth will vary – sometimes dramatically – along the sequence, and the mapping program used will wrongly align some of the reads. All of these effects mean that the number of nucleotides aligned to a specific position (if any) will vary, affecting the amount of information available to infer the genotype, and also that the aligned nucleotides might not all be homologous to the original position due to errors. If the organism is diploid and heterozygote at the position, we furthermore expect a random sampling from both alleles. We do not currently consider polyploidy in the framework.

**Figure 3:**
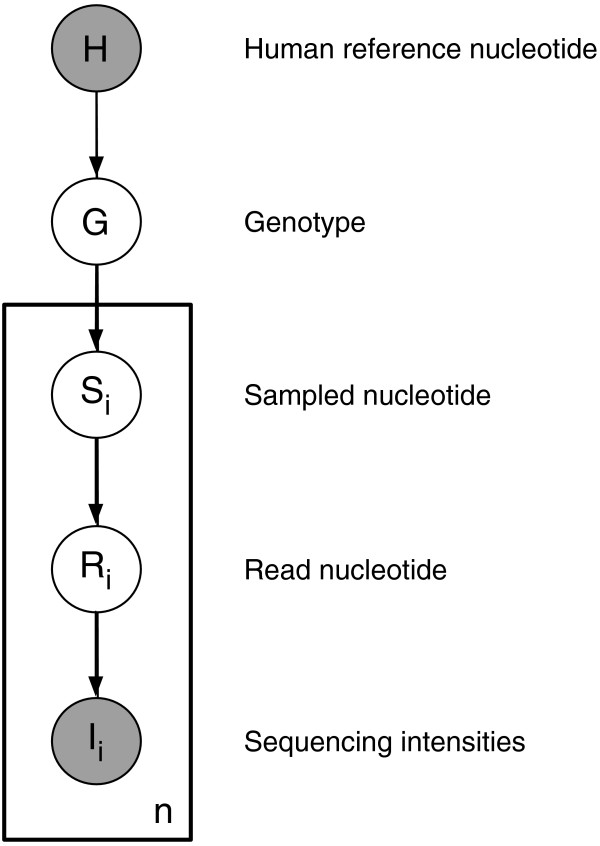
**The graphical model used in SNPest.** Circles represent random variables. The two top RVs are global for a given position, whereas the boxed part of the model denotes the *n* individual reads covering the specific position.

We developed SNPest to be a sensitive genotyping tool, designed to avoid systematic biases due to e.g. read errors. SNPest is a probabilistic model that takes quality scores – normally encoded using a Phred-like scheme [21] – and alternative sources of errors explicitly into account. It is based on a generative model of the probability distribution over genotypes given the sampling and sequencing of nucleotides obtained from a diploid genome as illustrated in Figure 3.

Let *i* indicate a specific read of the *n* possible reads covering a given position in the genome we are currently looking at. The observed random variables (RV) are the light intensities *I*_*i*_ and the reference nucleotide *H*. The unobserved RVs are the nucleotide in the read sequence *R*_*i*_, the originally sampled nucleotide *S*_*i*_, and the genotype in the diploid genome, *G*. For a given position in the genome, the combined probability distribution becomes:
1

The product is over the *n* reads aligned to the genomic position being analyzed, with variables *S*_*i*_, *R*_*i*_ and *I*_*i*_ corresponding to each of the reads. The probability factorizes into five separate probabilities: *P*(*H*) is the prior distribution over the reference nucleotide. *P*(*G*|*H*) is the conditional probability distribution over the ten possible genotypes {*AA*,*AC*,…,*TT*} given the reference nucleotide. *P*(*S*_*i*_|*G*) is the conditional probability distribution over the actual nucleotide present in the sampled DNA fragment given the genotype. *P*(*R*_*i*_|*S*_*i*_) is the conditional probability distribution over the read nucleotides given the actual nucleotide. *P*(*I*_*i*_|*R*_*i*_) is the conditional probability distribution over light intensities given the read nucleotide. The individual probability distributions are described in the following, including how the parameters can be estimated.

A haploid version of SNPest is obtained by changing the distribution over the genotypes to {*A*,*C*,*G*,*T*}, and *P*(*S*_*i*_|*G*) is simplified to be the identity matrix.

SNPest calls the genotype with the highest posterior probability at each position. This probability is found by marginalizing the combined probability distribution and summing out the *S* and *R* parameters. This gives the probability *P*(*G*,*I*,*H*), and by marginalizing and conditioning on *G* we can obtain *P*(*G*|*I*,*H*). The posterior probability of the genotype reflects the confidence that SNPest has in inferring that particular genotype. This information can be used to generate a high confidence subset of genotypes if needed.

The sequencing machines generally produce Phred-like quality scores together with the reads that provide the probability of error at each position. These qualities *Q* are given as a sequence of ASCII characters, where the actual quality scores *SC*(*Q*) are offset by some value *Δ*, *SC*(*Q*) = *ord*(*Q*)-*Δ* where *ord* gives the ASCII value of a character. The higher the quality score *SC*(*Q*), the lower the probability of an error:
2

SNPest uses both the reported read qualities, *Q*_*R*_, and the mapping qualities reported by the mapping tool used, *Q*_*M*_. When estimating the genotype, the smallest of the two is used: *Q* = *min*(*Q*_*R*_,*Q*_*M*_). Thus, reads mapped with low mapping quality (i.e. with small *Q*_*M*_ scores) will have a smaller weight in the calculations. Similarly, a low quality read, even if mapped with high certainty, will also have low weight in the calculations since it cannot betrusted.

### Reference nucleotide distribution

The model can either use a prior distribution on *P*(*H*), or it can be observed i.e. the nucleotide at the given position in the reference genome can be used. If the reference nucleotide is not used, the default behavior of SNPest is to use a flat prior over the four nucleotides. However, a user-specified distribution can be used instead if required.

### Genotype distribution

Given a nucleotide in the reference genome, the specimen being sequenced will have one of either 10 or 4 possible genotypes depending on whether it is a diploid or haploid genome. The distribution over genotypes *P*(*G*|*H*) could be estimated from a known genome (e.g. the Yanhuang genome [32]), or it can be estimated based on a known or expected SNP rate. In the simplest case, a flat prior could be used.

By default, SNPest assumes a SNP rate of . In the haploid case, the probability distribution simplifies to , i.e. the probability of the nucleotide being identical to the reference, and  for the three remaining cases, i.e. the probability of a SNP. This assumes a flat distribution and does not consider differences in transitions and transversions. However, such differences could be included in the model as was done in e.g. [33].

In the diploid case, the distribution over genotypes can be viewed as a 4×10 matrix with a row for each possible reference nucleotide describing the distribution over the possible genotypes. Let *P*(*H*=*N*) be the prior over the four nucleotides (by default, a flat prior of *P*(*H*=*N*)=0.25 is used). Further, let  be the expected SNP probability in the genome (again, a SNP rate of 0.1*%* is assumed by default). Then, for nucleotide *N*, the probability of observing the homozygote genotype, *NN*, becomes . The probability of a SNP is distributed over the remaining 9 possible genotypes. However, these 9 genotypes are not equally likely. For each nucleotide *N*, there are three genotypes that require just one alternative allele to be chosen, and six genotypes that require two alternative alleles. Assuming Hardy-Weinberg equilibrium, we express the probability distribution as follows: Let the probability of picking one alternative allele be *p*. Then the probability of the three genotypes requiring just one alternative allele becomes *p*(1-*p*) (i.e. the probability of picking an alternative allele times the probability of not picking an alternative), and the probability of the remaining six genotypes becomes *p*^2^. Solving this second degree equation yields the probabilities needed:


The probability of picking one alternative allele is *p*=0.0333*%*. As in the haploid case, this does not consider differences in transitions and transversions although that could be incorporated. SNPest comes with scripts to calculate the probability distribution given a specified SNP rate.

### Sampled nucleotide distribution

Given a genotype, we will be sampling one of the possible alleles with probability *P*(*S*_*i*_|*G*). In the haploid case, this collapses to the identity matrix. For a diploid genome there are two possible cases: If the genotype is homozygote in nucleotide *a*, then *P*(*S*_*i*_= *N*|*G*) = 100*%* for nucleotide *N* = *a* and *P*(*S*_*i*_= *N*|*G*) = 0*%* for the three other nucleotides, *N* ≠ *a*. If it is a heterozygote position, the probability is *P*(*S*_*i*_= *N*|*G*) = 50*%* for the two alleles and *P*(*S*_*i*_= *N*|*G*) = 0*%* for the remaining two.

### Read nucleotide distribution

In the ideal world, the nucleotide present in the DNA sequence being read will be identical to what was present in the original genome. However, due to various sources of error this will not always be the case. The probability distribution *P*(*R*_*i*_|*S*_*i*_) models all sources of error from the original DNA to the sequencing step e.g. from damage, PCR amplification errors, etc.

Calculating the length 4 vector  describing the nucleotide distribution *P*(*R*_*i*_|*I*_*i*_) directly from the quality scores (Eq. 2) assumes the quality measure is correct and perfectly reflects the errors in the data. However, this will not consider e.g. wrong mappings or other sources of error. The expected error distribution is given by *P*(*R*_*i*_|*S*_*i*_), and if we expect an error rate of *τ*, we multiply the vector  by an error matrix **A**, yielding the new probability vector .

The expected errors could be distributed uniformly over all the possible nucleotides. However, from counting actual wrong mappings in a haploid genome a pattern was observed showing that the error distribution depends on the nucleotide in the read: For sample nucleotide *A*, the highest probability for a read nucleotide is indeed *P*(*R* = *A*|*S* = *A*). However, it turns out that the second largest probability is *P*(*R* = *C*|*S* = *A*) = *P*(*R* = *G*|*S* = *A*), while the smallest probability is *P*(*R* = *T*|*S* = *A*). For the other three nucleotides, the smallest probabilities are, respectively, *P*(*R* = *G*|*S* = *C*), *P*(*R* = *C*|*S* = *G*) and *P*(*R* = *A*|*S* = *T*). In all cases, the smallest probability is approximately 1/3 of the second largest probability.

Using this observation and given an error rate *τ*, the error matrix mentioned above becomes:
3

By default, the error rate is estimated to be *τ* = 0.2*%*.

The DNA damage observed in ancient samples is clustered in the ends of the reads [25] and drops of exponentially. The amount of damage depends on various circumstances including the age of the sample and the environment in which it was found (e.g. temperature and humidity). The damage pattern is also observed in formalin-fixed and paraffin-embedded samples [11]. DNA damage leads to deamination of cytosine (C) into uracil (U), that will be reported as thymine (T) by the sequencer. The result is an excess of C to T mismatches. However, depending on the strand being sequenced the damage also leads to an excess of G to A mismatches. This means that if a *T* is observed in a read, there is a higher probability that it was a *C* in the sample, i.e. the probability *P*(*R* = *T*|*S* = *C*) should increase. Similarly, *P*(*R* = *A*|*S* = *G*) should increase. Given an overall expected damage rate, *δ*, an error matrix *A*_*δ*_ can be calculated by modifying Eq. 3:
4

The damage model used in SNPest uses and expected damage rate of *δ* = 3*%*. For computational reasons, the matrices describing errors, damage etc. are multiplied with the matrix describing the probability distribution *P*(*R*_*i*_|*I*_*i*_) prior to running the program. SNPest comes with scripts to calculate matrices given an expected error rate, 0≤*τ* < 1, and an expected damage rate, 0 ≤ *δ* < 1. Setting both *τ* = 0 and *δ* = 0 means that the reported quality scores are trusted completely and used directly when estimating genotypes.

### Distribution of light intensities

This distribution models the probability of observing a specific light intensity given the nucleotide in the read. We do not know the form of the conditional probability distribution *P*(*I*_*i*_|*R*_*i*_). However, this probability is proportional to *P*(*R*_*i*_|*I*_*i*_) which is given by the quality scores (see Eq. 2), when we assume *P*(*R*_*i*_) is equal for all *R*_*i*_, i.e., that all four nucleotides have equal prior probability. Note that SNPest does not use the actual light intensities and does not require anything but the fastq files with quality scores. From this, the posterior probability is calculated as explained above.

### Program input and output

SNPest takes input generated by the “samtools mpileup -s” command (or equivalent) reporting nucleotides mapped to each position together with read qualities and mapping qualities. The output is in VCF format with a line per position covered in the genome. We provide a Perl script for parsing this output to produce a smaller VCF file with only high quality SNPs and insertions/deletions (as defined by the user based on minimum depth, quality scores etc.). The output contains information on the reference nucleotide (if given), the most likely genotype, the posterior probability (in Phred quality format), read depth used, and average mapping quality. Please refer to the README file provided with the program for more details on how to run SNPest.

## Availability and requirements

**Project name:** SNPest**Project home page:**https://github.com/slindgreen/SNPest**Operating system:** Tested on Unix and MacOS**Programming language:** C++ and PERL**Other requirements:**https://github.com/jakob-skou-pedersen/phy**License:** GNU GPL 3

## Electronic supplementary material

Additional file 1: **Supplementary material for SNPest: A probabilistic graphical model for estimating genotypes.** (PDF 534 KB)
