# Supplementary material for: SNPest: a probabilistic graphical model for estimating genotypes
Source: BMC Res Notes. 2014 Oct 7;7:698. doi: 10.1186/1756-0500-7-698 (PMC4203901; doi:10.1186/1756-0500-7-698)
Supplement: Supplementary file 1 — Additional file 1: Supplementary material for SNPest: A probabilistic graphical model for estimating genotypes. (PDF 534 KB) [file 13104_2014_3244_MOESM1_ESM.pdf]

Supplementary material for SNPest: A probabilistic graphical model for estimating genotypes

Stinus Lindgreen [1,2,3]*, Anders Krogh [1,2] and Jakob Skou Pedersen [1,4]

July 6, 2014

[1]Section for Computational and RNA Biology, Department of Biology, University of Copenhagen, Ole Maaloes Vej 5, 2200 Copenhagen, Denmark
[2]Center of Excellence for GeoGenetics, Natural History Museum of Denmark and Department of Biology, University of Copenhagen, Oester Voldgade 5-7, 1350 Copenhagen, Denmark
[3]School of Biological Sciences, University of Canterbury, Private Bag 4800, Christchurch 8041, New Zealand
[4]Department of Molecular Medicine, Aarhus University Hospital, Skejby, Brendstrupgaardsvej 100, DK-8200 Aarhus N, Denmark

1 Haploid data

The data sets used and the different mapping programs and parameters are presented in the main text. Below are additional results produced in the test.

1.1 *E. coli*, adapters removed

The cleaned *E. coli* data was analyzed using the haploid mode of SNPest both with and without using the reference genome.

Table 1: *E. coli*, adapters removed, SNPest used with haploid model, reference genome ignored.

	Bowtie2		BWA-PSSM	
Depth	All	QC	All	QC
5	41	2	92	4
10	6	0	45	0
20	6	0	44	0
30	6	0	44	0
40	6	0	44	0
50	6	0	44	0
60	6	0	44	0

*to whom correspondence should be addressed: stinus@binf.ku.dk

1.2 *E. coli*, adapters present

The *E. coli* data was analyzed without removing adapter sequences. The haploid model was used, and SNPest was run both with and without using the reference genome. GeMS and FreeBayes were run using the haploid setting.

Table 2: *E. coli*, adapters present, SNPest used with haploid model.

	Reference genome used				Reference genome ignored			
	Bowtie2		BWA-PSSM		Bowtie2		BWA-PSSM	
Depth	All	QC	All	QC	All	QC	All	QC
5	30	1	28	3	181	40	178	25
10	3	1	0	0	22	5	30	3
20	1	0	0	0	6	0	24	0
30	0	0	0	0	6	0	24	0
40	0	0	0	0	6	0	24	0
50	0	0	0	0	6	0	24	0
60	0	0	0	0	6	0	24	0

Table 3: *E. coli*, adapters present, GeMS used with haploid model.

	Bowtie2		BWA-PSSM	
Depth	All	QC	All	QC
5	8	2	22	5
10	5	0	18	2
20	5	0	18	2
30	5	0	18	2
40	5	0	18	2
50	5	0	18	2
60	5	0	18	2

Table 4: *E. coli*, adapters present, FreeBayes used with haploid model.

Bowtie2		BWA-PSSM	
All	QC	All	QC
534	2	311	0

1.3 *E. coli*, simulated ancient DNA

Ancient DNA was simulated based on the *E. coli* genome as described in the main text. The data was analyzed using the haploid model, with the general error model and the specific damage model, and using the reference genome.

Table 5: Simulated ancient DNA from *E. coli*, SNPest used with haploid model, reference genome used.

	General error model				Specific damage model			
	Bowtie2		BWA-PSSM		Bowtie2		BWA-PSSM	
Depth	All	QC	All	QC	All	QC	All	QC
5	12	0	45	4	1	0	6	0
10	5	0	12	0	0	0	2	0
20	5	0	12	0	0	0	2	0
30	5	0	12	0	0	0	2	0
40	5	0	12	0	0	0	2	0
50	5	0	12	0	0	0	2	0
60	5	0	12	0	0	0	2	0

2 Diploid data from human

Data from the 1000 Genome Project was used as described in the main text. Below are the results when using the reference genome (see main text for results without using the reference genome).

Table 6: Results on low depth, diploid data from human chromosome 20. The results from SNPest (using the reference genome), FreeBayes, SAMtools with bcftools, GATK's HaplotypeCaller and GeMS are shown. For each method, we report the number of high quality SNPs, the SNP rate, the fraction overlap with dbSNP 139, the fraction of SNPest predictions in common, the fraction of exclusive SNPs only predicted by this method, number of insertions/deletions, fraction of insertions/deletions found in dbSNP 139, and homozygous:heterozygous ratio for SNPs.

Program	#SNPs	SNP rate	dbSNP	SNPest	Excl.	Indels	dbSNP	Homo:hetero
SNPest	9,371	0.02%	99.08%	100.00%	0.10%	454	59.03%	0.00
FreeBayes	3,175	0.01%	98.90%	4.92%	2.58%	330	60.91%	1.12
SAMtools	65,120	0.11%	99.01%	99.48%	1.76%	6,918	60.18%	0.66
GATK	54,441	0.09%	99.44%	97.10%	1.18%	7,773	60.77%	1.09
GeMS	73,694	0.13%	87.59%	95.92%	17.95%	N/A	N/A	0.62

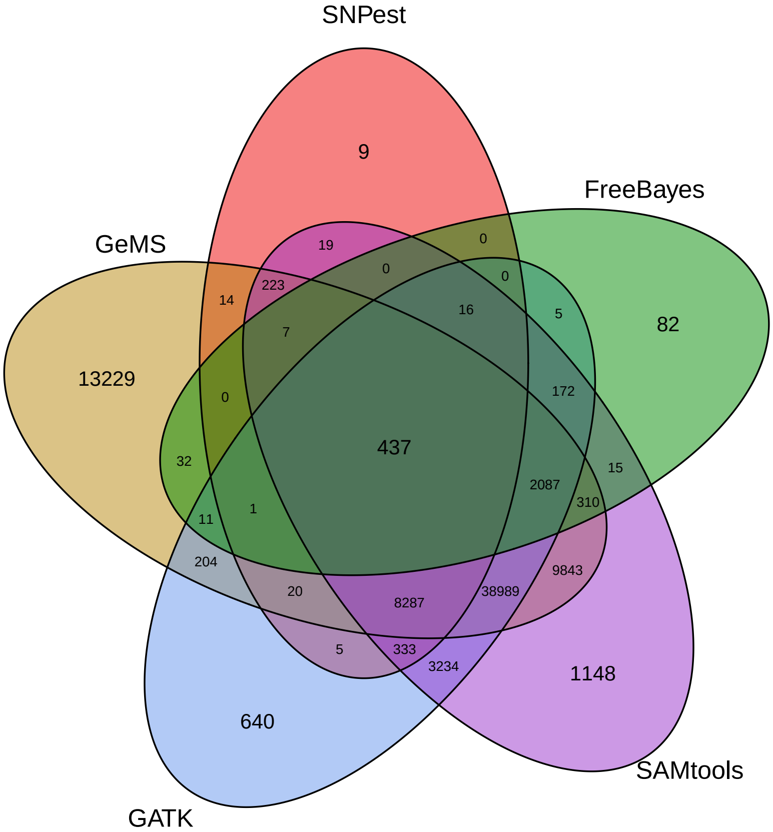

Figure 1: Predicted SNPs on low depth diploid data. The Venn diagram illustrates the performance of SNPest (using the reference genome in the calculations), GeMS, GATK, SAMtools and FreeBayes on diploid data from human chromosome 20.

Table 7: Results on high depth, diploid data from human chromosome 22. The results from SNPest (using the reference genome), FreeBayes, SAMtools with bcftools, GATK's HaplotypeCaller and GeMS are shown. For each method, we report the number of high quality SNPs, the SNP rate, the fraction overlap with dbSNP 139, the fraction of SNPest predictions in common, the fraction of exclusive SNPs only predicted by this method, number of insertions/deletions, fraction of insertions/deletions found in dbSNP 139, and homozygous:heterozygous ratio for SNPs.

Program	#SNPs	SNP rate	dbSNP	SNPest	Excl.	Indels	dbSNP	Homo:hetero
SNPest	37,362	0.11%	99.16%	100.00%	0.76%	82	57.32%	0.31
FreeBayes	11,570	0.03%	87.99%	14.44%	29.57%	511	60.86%	4.67
SAMtools	43,679	0.13%	99.37%	97.04%	0.41%	3,880	57.45%	0.49
GATK	43,721	0.13%	99.29%	95.47%	2.85%	5,660	56.29%	0.58
GeMS	51,117	0.15%	89.22%	97.72%	13.71%	N/A	N/A	0.50

3 Command lines

3.1 SNPest

SNPest was run on the many different haploid data sets using the following command line:

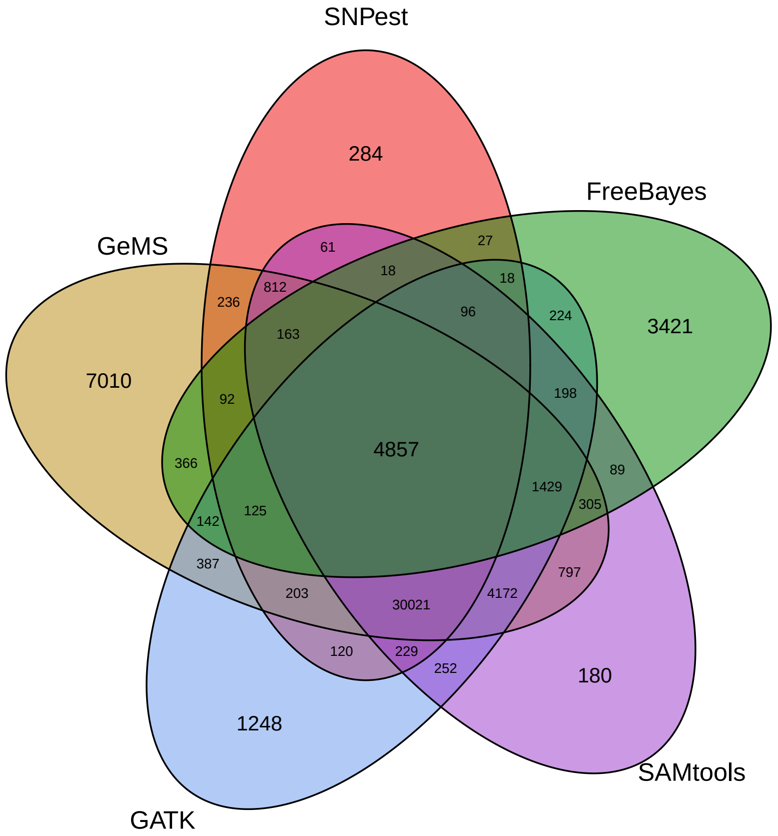

Figure 2: Predicted SNPs on high depth diploid data. The Venn diagram illustrates the performance of SNPest (using the reference genome in the calculations), GeMS, GATK, SAMtools and FreeBayes on diploid data from human chromosome 22.

Table 8: Comparison of predicted SNPs on chr20 between all pairs of methods. SNPest is run without using the reference genome as a prior. Each row shows the percentage of predictions for that method that overlaps with the methods in each column.

Chr 20	SNPest, no ref	FreeBayes	SAMtools	GATK	GeMS
SNPest, no ref	100.00%	5.56%	99.46%	97.84%	99.24%
FreeBayes	24.79%	100.00%	95.87%	85.95%	90.87%
SAMtools	21.62%	4.67%	100.00%	82.24%	92.42%
GATK	25.45%	5.01%	98.37%	100.00%	91.91%
GeMS	19.07%	3.91%	81.67%	67.90%	100.00%

```
cat [mpileup file] | SNPest_static.pl --execpath=/share/data/bin/phy/src
                   --ploidity=haploid --maxdepth=[depth] --model=[model]
                   [reference fasta] | bzip2 > [basename].full.vcf.bz2
bzcat [basename].full.vcf.bz2 | cleanupvcf.pl > [basename].clean.vcf
```

The specific settings depend on the actual data set:

Table 9: Comparison of predicted SNPs on chr20 between all pairs of methods. SNPest is run using the reference genome as a prior. Each row shows the percentage of predictions for that method that overlaps with the methods in each column.

Chr 20	SNPest, use ref	FreeBayes	SAMtools	GATK	GeMS
SNPest, use ref	100.00%	4.92%	99.48%	97.10%	95.92%
FreeBayes	14.52%	100.00%	95.87%	85.95%	90.87%
SAMtools	14.32%	4.67%	100.00%	82.24%	92.42%
GATK	16.71%	5.01%	98.37%	100.00%	91.91%
GeMS	12.20%	3.91%	81.67%	67.90%	100.00%

Table 10: Comparison of predicted SNPs on chr20 between all pairs of methods. SNPest is run without using the reference genome as a prior. Each row shows the percentage of predictions for that method that overlaps with the methods in each column.

Chr 22	SNPest, no ref	FreeBayes	SAMtools	GATK	GeMS
SNPest, no ref	100.00%	14.52%	96.92%	95.40%	97.78%
FreeBayes	51.45%	100.00%	61.84%	61.27%	64.64%
SAMtools	90.97%	16.38%	100.00%	94.45%	97.43%
GATK	89.46%	16.21%	94.36%	100.00%	94.54%
GeMS	78.42%	14.63%	83.25%	80.87%	100.00%

Table 11: Comparison of predicted SNPs on chr20 between all pairs of methods. SNPest is run using the reference genome as a prior. Each row shows the percentage of predictions for that method that overlaps with the methods in each column.

Chr 22	SNPest, use ref	FreeBayes	SAMtools	GATK	GeMS
SNPest, use ref	100.00%	14.44%	97.04%	95.47%	97.72%
FreeBayes	46.64%	100.00%	61.84%	61.27%	64.64%
SAMtools	83.01%	16.38%	100.00%	94.45%	97.43%
GATK	81.58%	16.21%	94.36%	100.00%	94.54%
GeMS	71.42%	14.63%	83.25%	80.87%	100.00%

- mpileup file is a file containing the output from the aligned bam-file using the command line: `samtools mpileup -s -q 25 -Q 0 -f [reference fasta] [bam file]`
- depth sets the maximum read depth used (i.e. varied from 5 to 60).
- model describes the specific model used (e.g damage, error, none).
- reference fasta is the fasta file containing the reference genome.
- basename is the filename used for the output for the specific test run.

For diploid data, a similar command line was used except that the model was always "error", the ploidity was always "diploid", and the maximum depth was always "200".

3.2 GeMS version 1.0

For the haploid test sets the following command line was used:

```
cat [mpileup file] | gems -m [depth] -d 1 -o [basename]
```

The parameters "mpileup file", "depth" and "basename" are defined as above. The parameter "-d 1" means haploid data.

For the diploid test, a similar command was used but "-m 255" (the default) was used, and parameter "-d 0" was used to indicate diploid data.

3.3 FreeBayes version v9.9.2-14-g3e07445-dirty

The haploid tests were run with the following command

```
freebayes -p 1 -b [bam file] -f [reference fasta] -v [basename].FreeBayes.total.vcf
```

The parameters "bam file", "reference fasta" and "basename" are defined above. The diploid test is run using a similar command but setting "-p 2" to indicate diploid data.

3.4 SAMtools/bcftools

SAMtools (version 0.1.18 (r982:295)) and bcftools (version 0.1.17-dev (r973:277)) were only run on the diploid data using the following command:

```
samtools mpileup -uf [reference fasta] [bam file] | bcftools view -bvcg - >
     [base name].SAMtools_bcftools.total.bcf
bcftools view [base name].SAMtools_bcftools.total.bcf |
     vcfutils.pl varFilter -D100 > [base name].SAMtools_bcftools.QC.vcf
```

3.5 GATK version v3.1-1

GATK's haplotypecaller was run using the following commands:

```
PATH_TO_GENOMEANALYSISTK=/com/extra/GATK/LATEST/jar-bin
REF_GENOME=/home/vang/reference/human_g1k_v37.fasta/human_g1k_v37.fasta
HEAP=40
DBSNP=/home/vang/reference/human_g1k_v37.fasta/dbsnp_138.b37.vcf
ORIGINAL_BAMFILE=[bam file]
DIRBAM=‘dirname ${ORIGINAL_BAMFILE}‘
BASEBAM=‘basename ${ORIGINAL_BAMFILE}‘
java -Xmx${HEAP}g -jar ${PATH_TO_GENOMEANALYSISTK}/GenomeAnalysisTK.jar
     -T HaplotypeCaller -nct 16 -R ${REF_GENOME} -L 20 -I ${ORIGINAL_BAMFILE}
     --genotyping_mode DISCOVERY --dbsnp ${DBSNP} -stand_emit_conf 10
     -stand_call_conf 30 -minPruning 3 -o ${ORIGINAL_BAMFILE%.bam}.HaplotypeCaller.vcf
     --log_to_file ${ORIGINAL_BAMFILE%.bam}.HaplotypeCaller.error.log
```

Where "bam file" is one of the two diploid test sets used. Note that the concordance with dbSNP was done afterward using dbSNP 139. Also, note that the option '-minPruning 3' was recommended when the test was run, but now the recommended setting is '-minPruning 2'.
